# Early-onset metastatic fibroblastic osteosarcoma of the metatarsus in a young cat: a case report

**DOI:** 10.1186/s12917-025-05223-8

**Published:** 2025-12-29

**Authors:** Mojtaba Kiakojoori, Hossein Kazemi Mehrjerdi, Ali Mirshahi, Mahdieh Zaeemi, Mohsen Maleki

**Affiliations:** 1 Department of Clinical Sciences, Faculty of Veterinary Medicine, Ferdowsi University of Mashhad, Mashhad, Iran; 2 Department of Pathobiology, Faculty of Veterinary Medicine, Ferdowsi University of Mashhad, Mashhad, Iran

**Keywords:** Fibroblastic osteosarcoma, Metatarsus, Radiology, Histopathology, Cat

## Abstract

**Background:**

Osteosarcoma is the most common primary bone tumor in cats, yet it is typically diagnosed in older animals and rarely metastasizes. Among its histological variants, the fibroblastic subtype—defined by spindle-shaped osteogenic cells that generate osteoid—is infrequently reported in young cats.

**Case presentation:**

A one-year-old intact female domestic shorthair (DSH) cat was referred to the Veterinary Teaching Hospital of Ferdowsi University of Mashhad with a one-month history of progressive lameness and a firm swelling on the right hindlimb. Radiographs revealed an aggressive osteolytic lesion of the fourth metatarsal bone with a Codman’s triangle periosteal reaction, as well as a pulmonary mass consistent with metastasis. Fine-needle aspiration cytology demonstrated pleomorphic osteoblasts with anisokaryosis, multinucleated giant cells, and osteoid material, suggesting osteosarcoma. Following humane euthanasia, necropsy revealed extensive bone destruction and metastatic foci in the lung and popliteal lymph node. Histopathology showed a mixture of spindle-shaped and pleomorphic osteogenic cells producing eosinophilic osteoid matrix, confirming a diagnosis of fibroblastic osteosarcoma. Immunohistochemistry showed strong nuclear positivity for SATB2, confirming osteoblastic lineage. Hepatic and splenic changes were reactive and non-metastatic.

**Conclusions:**

This report describes an exceptionally early-onset fibroblastic osteosarcoma in a one-year-old cat, accompanied by both pulmonary and lymphatic metastasis, a presentation rarely reported in feline patients. Recognition of such atypical presentations broadens the understanding of feline osteosarcoma behavior and underscores the value of integrating radiologic, cytologic, histopathologic, and immunohistochemical findings for accurate diagnosis.

## Background

Primary bone tumors in cats are uncommon, with an estimated incidence of 4.9 per 100,000 cats. Among these, 67% to 90% are histologically classified as malignant. Osteosarcomas are the most prevalent primary bone tumor in this species, accounting for approximately 70% to 80% of all primary malignant bone neoplasms in cats. The predominant clinical manifestations of appendicular osteosarcoma in felines are lameness, edema, and deformity, depending on the lesion’s location. Radiographically, feline osteosarcoma resembles canine osteosarcoma, exhibiting mixed osteoblastic and osteolytic alterations and an indistinct transition zone between normal and cancerous bone. However, juxtacortical osteosarcoma has also been documented in cats. Tumors may attain significant dimensions without manifesting severe clinical symptoms. Metastasis at presentation is uncommon in cats [1]. Feline osteosarcomas generally exhibit a more indolent progression and are less likely to metastasize, with a metastatic rate of 5% to 10% [1–3]. This starkly contrasts with its behavior in dogs, where the disease is aggressive and prone to rapid metastasis [4]. Amputation is the preferred treatment for non-metastatic appendicular osteosarcoma in cats. The administration of adjuvant chemotherapy is neither indicated nor recommended in feline cases [1].

Osteosarcomas can be categorized into histological subtypes, including osteoblastic, chondroblastic, fibroblastic, telangiectatic, poorly differentiated, nonproductive, productive, and giant cell types, each distinguished by specific cellular characteristics [5]. Among these, the fibroblastic osteosarcomas often arise as lytic lesions; however, roughly half eventually display a mixed radiographic pattern as the neoplastic spindle cells gain increased capacity to produce mineralized bone matrix [5]. 

Several atypical features distinguish our case: the patient’s exceptionally young age, the rapid progression of metastatic disease, and the tumor’s unusual distal metatarsal origin. Such a constellation of findings is rarely documented in cats, in whom osteosarcoma typically arises in older animals and exhibits limited metastatic potential. Consequently, this report contributes new insight into the range of biological behaviors and anatomical presentations possible in feline osteosarcoma.

## Case presentation

**Fig. 1:**
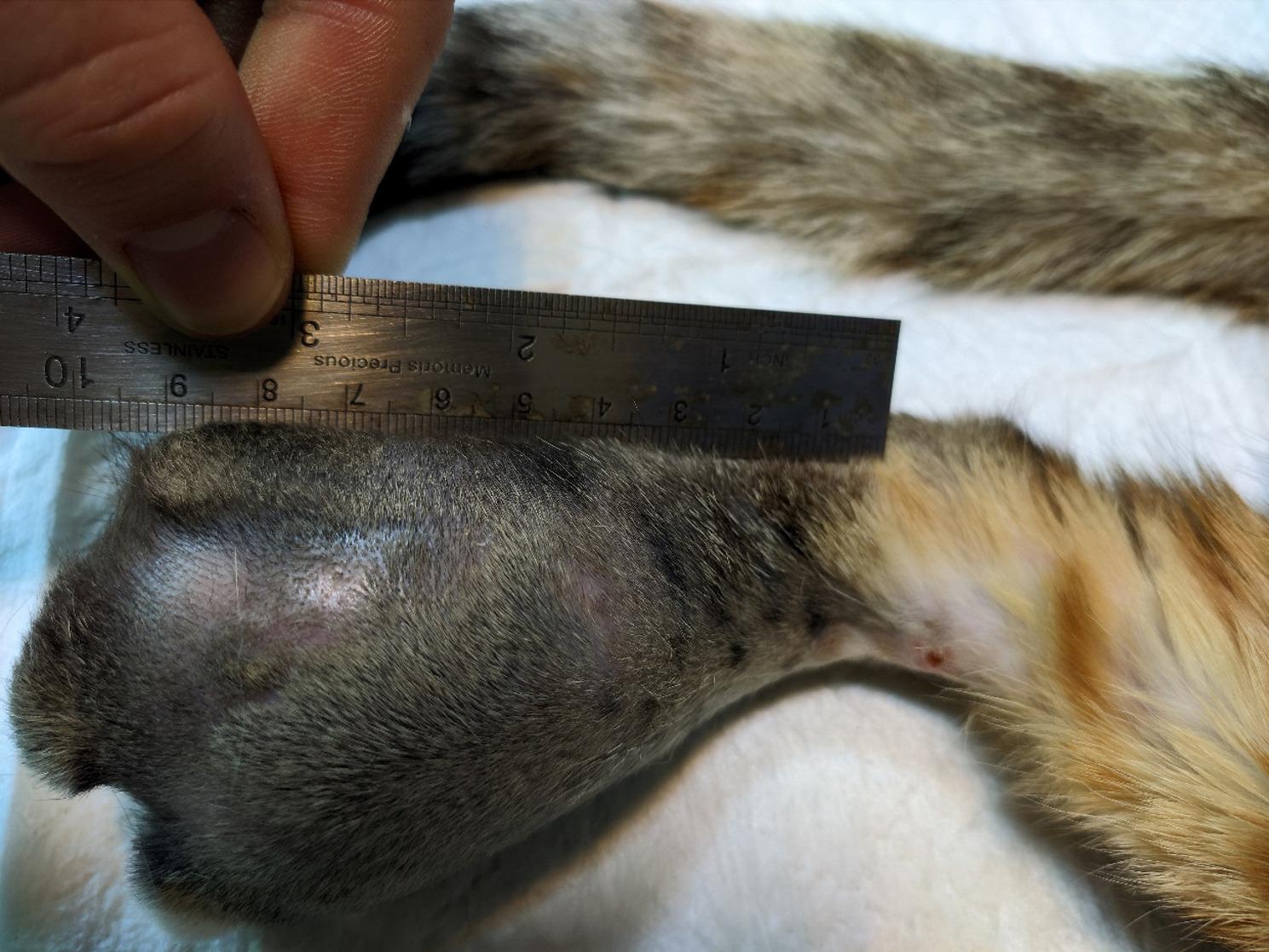
Macroscopic view of the metatarsal mass

**Fig. 2:**
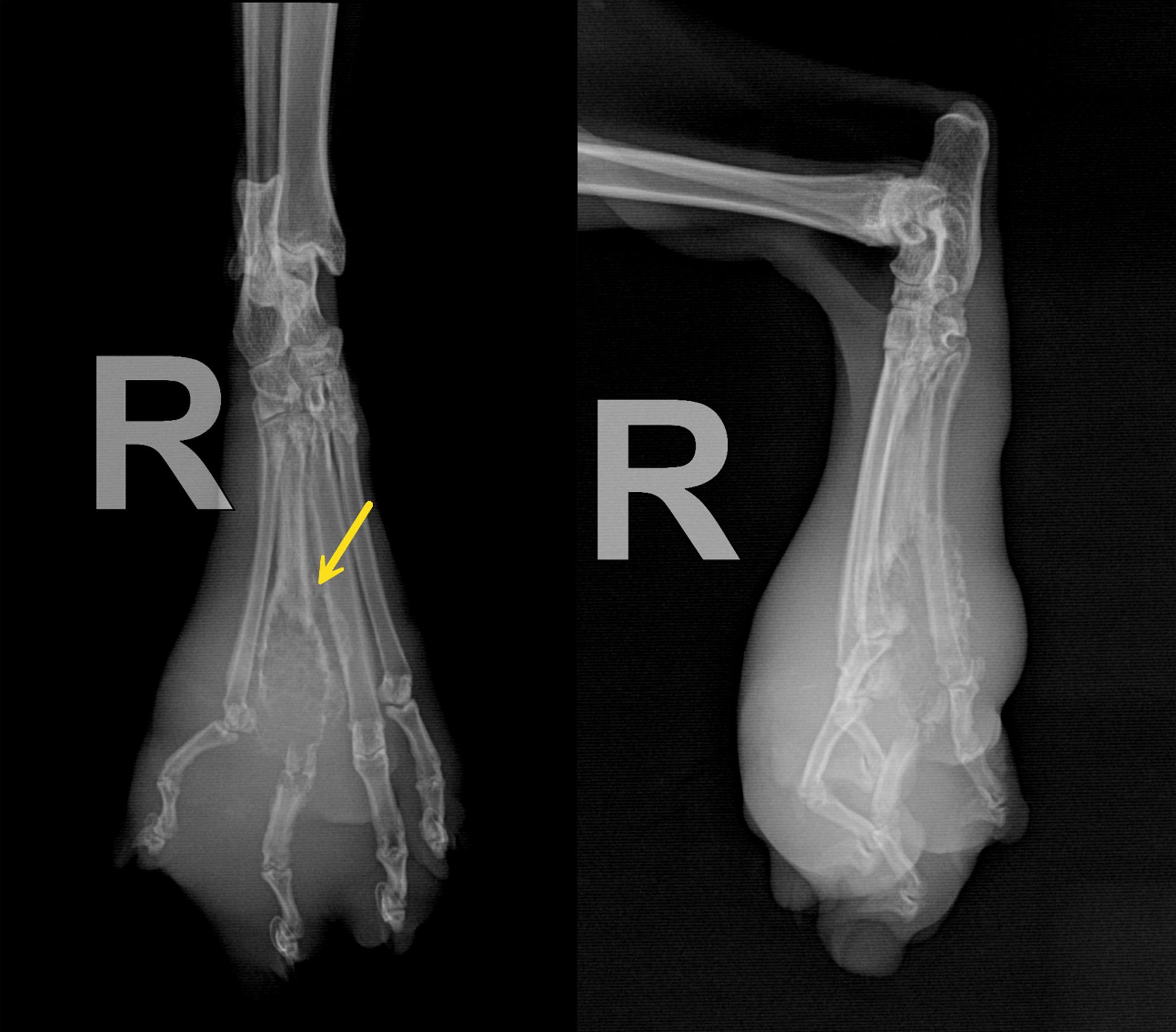
Lateral and dorsoplantar radiographs of the right distal hindlimb. Aggressive bone reactions, moth-eaten, permeative osteolysis, cortical destruction, and Codman’s triangle periosteal reaction (Yellow arrow) were seen in the 4th metatarsal bone

**Fig. 3:**
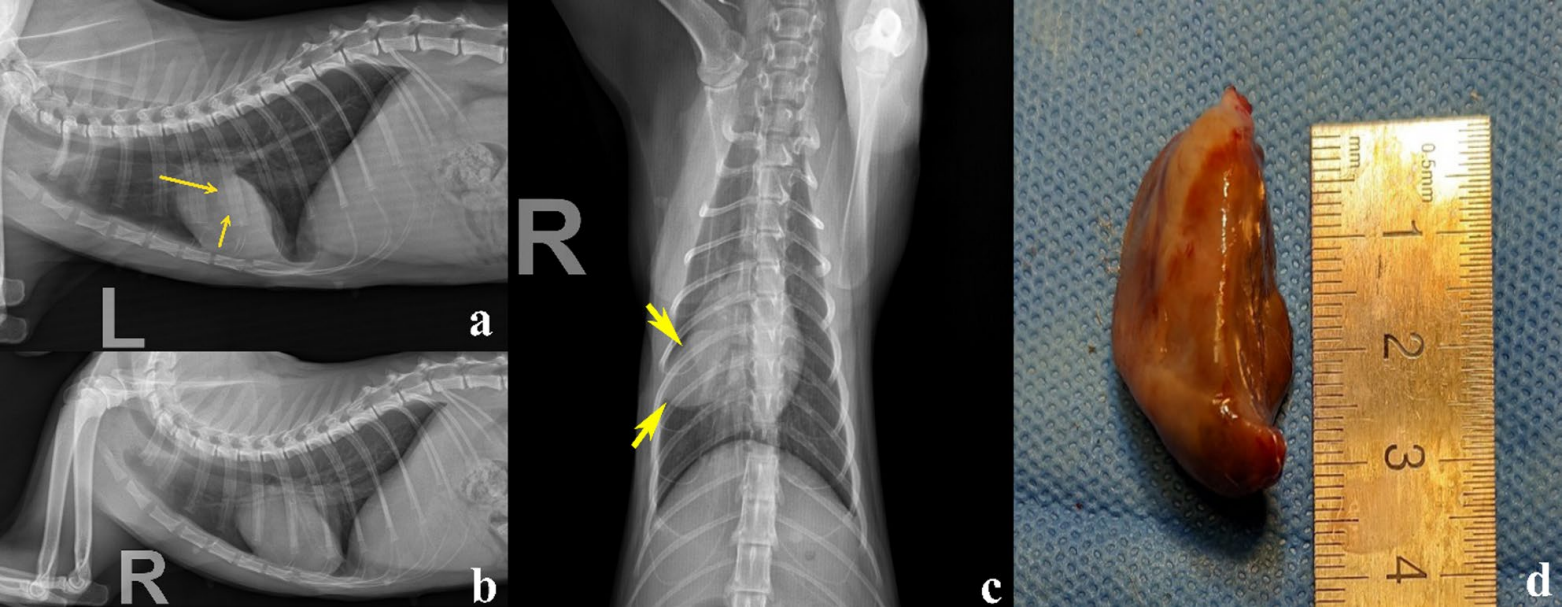
(**a** and **b**) Left and right lateral radiographs of the chest. The solitary metastatic mass lesion (nodular pattern) can be seen in the left lateral radiograph (Yellow arrows). (**c**) Ventrodorsal radiograph of the chest. Yellow arrows show the margins of the metastatic mass. (**d**) Metastatic mass in the right lung

**Fig. 4:**
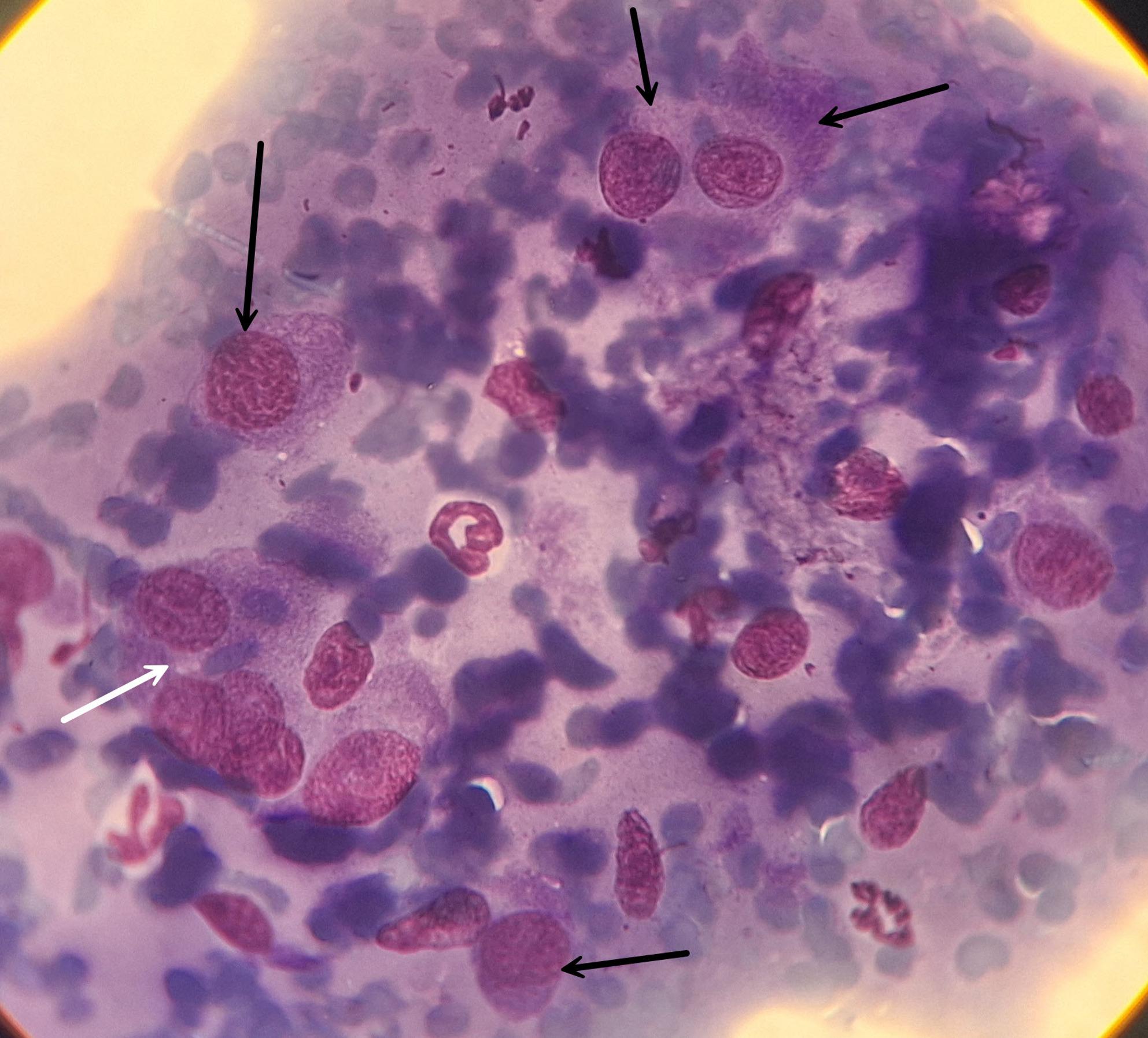
The cytological smear of osteosarcoma shows osteoblasts with eccentrically placed nuclei and prominent nucleoli (black arrows) and an osteoclast with multiple, relatively uniform nuclei and abundant cytoplasm (white arrows). Giemsa stain 1000X

A one-year-old intact female Domestic Shorthair (DSH) cat weighing 3 kg was referred to the Veterinary Teaching Hospital of Ferdowsi University of Mashhad, Iran, with a firm mass on its right foot. The owner stated that the cat had exhibited lameness over the past month and that the mass had grown rapidly over the last week. The owner’s chief complaint was anorexia, lethargy, progressive lameness, and a growing hindlimb mass on the metatarsal region. The mass was painful to touch and measured approximately 6 × 4 × 10 cm (Fig. 1). The popliteal lymph node of the same limb was also swollen. No other palpable masses were present. A Complete Blood Cell count (CBC) and full biochemistry profile were performed (Table 1). The biochemical profile showed hypercholesterolemia, mildly elevated serum alkaline phosphatase (ALP) activity, mild hypochloremia, mild hyponatremia, and hyperkalemia. Hematological indices, such as Packed Cell Volume (PCV), Red Blood Cells (RBC), Mean Corpuscular Hemoglobin (MCH), and Mean Corpuscular Volume (MCV), were at the lower limit of the reference interval, while the hemoglobin concentration was below normal values, indicating anemia in this case. In the radiographic examination, lateral and dorsoplantar views of the right distal hindlimb were taken (Fig. 2). There was severe soft tissue swelling in the right distal limb, particularly around the 4th metatarsal bone and 4th digit, with osteolytic, expansile bony reactions. The lesion had caused marked thinning of the cortex, and as a result, the cortex had been eroded. Codman’s triangle periosteal reaction was seen in the 4th metatarsal bone and proximal phalanx of the 4th digit. Radiographic findings confirmed an aggressive bone lesion. Left and right lateral and Ventrodorsal (VD) views of the thoracic region were taken to evaluate metastatic lesions. A soft tissue mass measuring approximately 3 × 2 cm was seen in the right middle lung lobe (Fig. 3). Additionally, a diffuse, mild bronchointerstitial pattern was observed in all lung lobes, suggesting pneumonia, bronchopneumonia, bronchitis, or pulmonary edema. An abdominal ultrasound was performed to detect possible metastatic lesions. No abnormal masses were detected in the abdominal cavity, and the liver was slightly hypoechoic. Fine Needle Aspiration (FNA) was performed on the metatarsal mass using a 2 mL syringe and a 23-gauge needle. Cytological evaluation of the metatarsal lesion revealed numerous atypical osteoblasts varying from round to fusiform with anisocytosis, anisokaryosis, and multiple prominent nucleoli, basophilic cytoplasm, and occasionally eosinophilic granules. Upon low-power examination of the slide, islands of osteoid, fibrillar, and bright-pink material were observed, surrounded by tumor cells (Fig. 4). Some osteoclasts with multiple, relatively uniform nuclei and abundant cytoplasm with eosinophilic stippling were observed in the smears. Osteoclasts are large and irregularly shaped with a variable number of uniform, round nuclei arranged randomly throughout the cell and abundant, light blue cytoplasm. Because the cytological analysis and radiology results indicated osteosarcoma with possible metastasis, we suggested euthanasia, which the owner accepted. Euthanasia was elected after thorough discussion with the owner due to the cat’s rapidly deteriorating condition, severe pain unresponsive to multimodal analgesia, and poor prognosis. Radiographic and cytologic examinations revealed extensive osteolysis of the metatarsus with pulmonary and lymphatic metastases, indicating advanced, non-resectable disease. Considering the animal’s compromised welfare and the lack of feasible therapeutic options, the cat was humanely euthanized via intravenous administration of an overdose of sodium thiopental under general anesthesia, in accordance with the American Veterinary Medical Association (AVMA) Guidelines for the Euthanasia of Animals [8], and a necropsy was performed with the owner’s consent. While examining the metatarsal mass, severe bone destruction and callus formation were observed. Samples from the remains of the fourth metatarsal bone were taken and preserved in buffer 10% formalin. The Macroscopic examination showed that the popliteal lymph node of the affected limb was somewhat enlarged, and the liver was slightly icteric, resembling hepatic lipidosis. Samples were taken from both tissues. Other internal organs of the abdominal cavity were unremarkable. Additional samples were taken from the spleen to check for possible metastasis. Then, the thoracic cavity was opened, and a 3 × 2 cm mass was observed in the right lung, adjacent to the heart, with considerable vascularization (Fig. 3). Samples from this mass were also preserved in buffer 10% formalin and sent to the lab.

**Table 1:** CBC and biochemistry profile. The reference values for hematology and biochemistry profiles were obtained from schalm’s veterinary hematology and clinical biochemistry of domestic Animals, respectively [6, 7]

Hematology					
		Reference range			Reference range
PCV (%)	24.5	24–45	Total WBC (/µL)	13,700	5500–19,500
HB (g/dL)	7.7	8–15	Neutrophil (/µL)	9864	2500–12,500
RBC (×10^6^/µL)	5.99	5–10	Eosinophil (/µL)	274	0–1500
MCV (fL)	40.9	39–55	Lymphocyte (/µL)	3425	1500–7000
MCH (pg)	12.9	12–15.8.8	Monocyte (/µL)	137	0–850
MCHC (g/dL)	31.4	31–35	Platelets (×10^3^/µL)	89	87–610
RDW (%)	14.9	24.9–40.6			

Biochemistry					
		Reference range			Reference range
Cholesterol (mg/dL)	185	95–130	ALP (U/L)	159.6	25–93

*PCV* Packed cell volume, *HB* Hemoglobin, *RBC* Red blood cell, *MCV* Mean corpuscular volume, *MCHC* Mean corpuscular hemoglobin concentration, *RDW* Red cell distribution width, *WBC* White blood cell, *ALP* Alkaline phosphatase

**Fig. 5:**
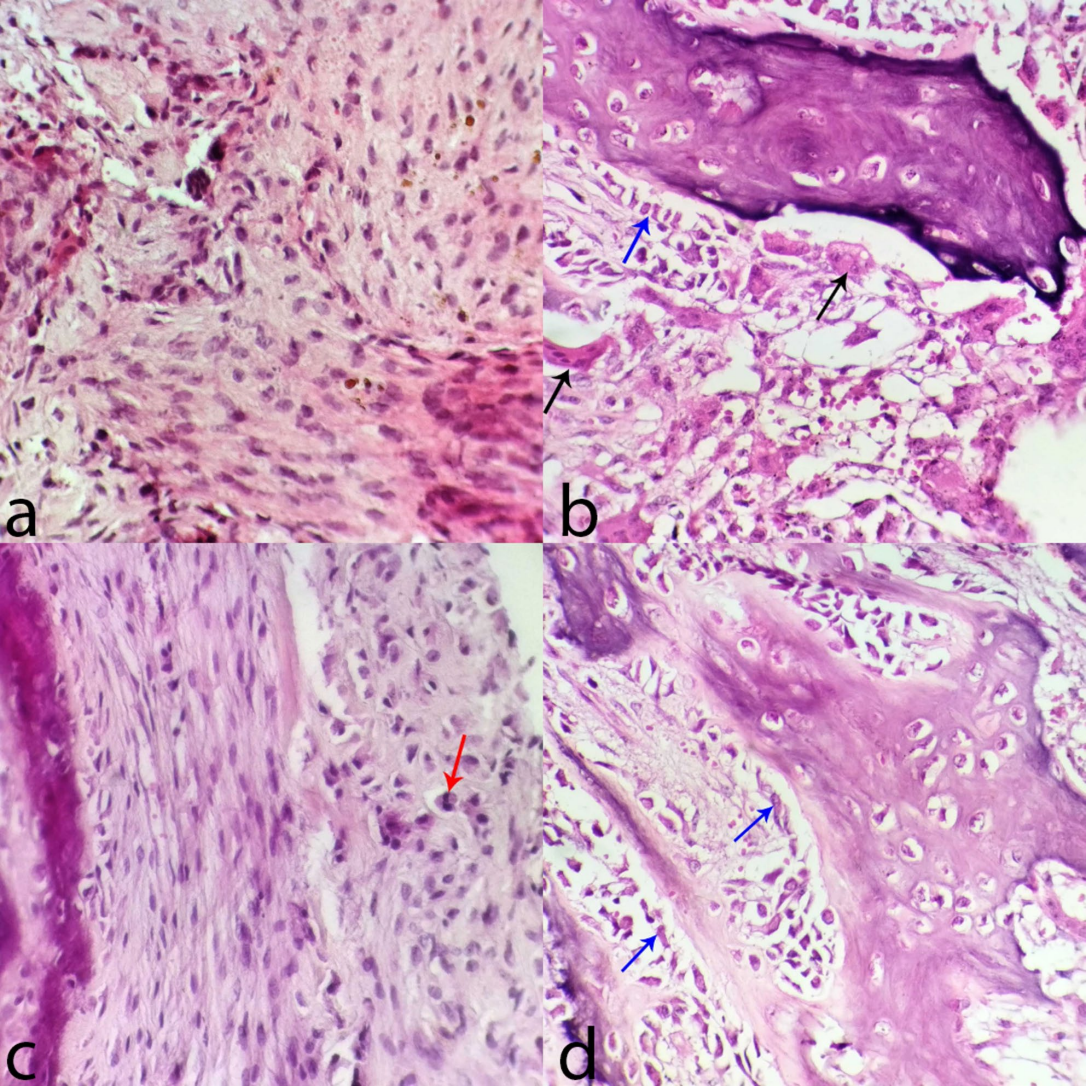
Photomicrographs of the metatarsal bone. **a**, Abnormal spindle-shaped cells. **b **&** d**, black arrow: giant cell, blue arrow: hyperactive osteoblast. **c**, Mitotic figure. H&E stain 400X

**Fig. 6:**
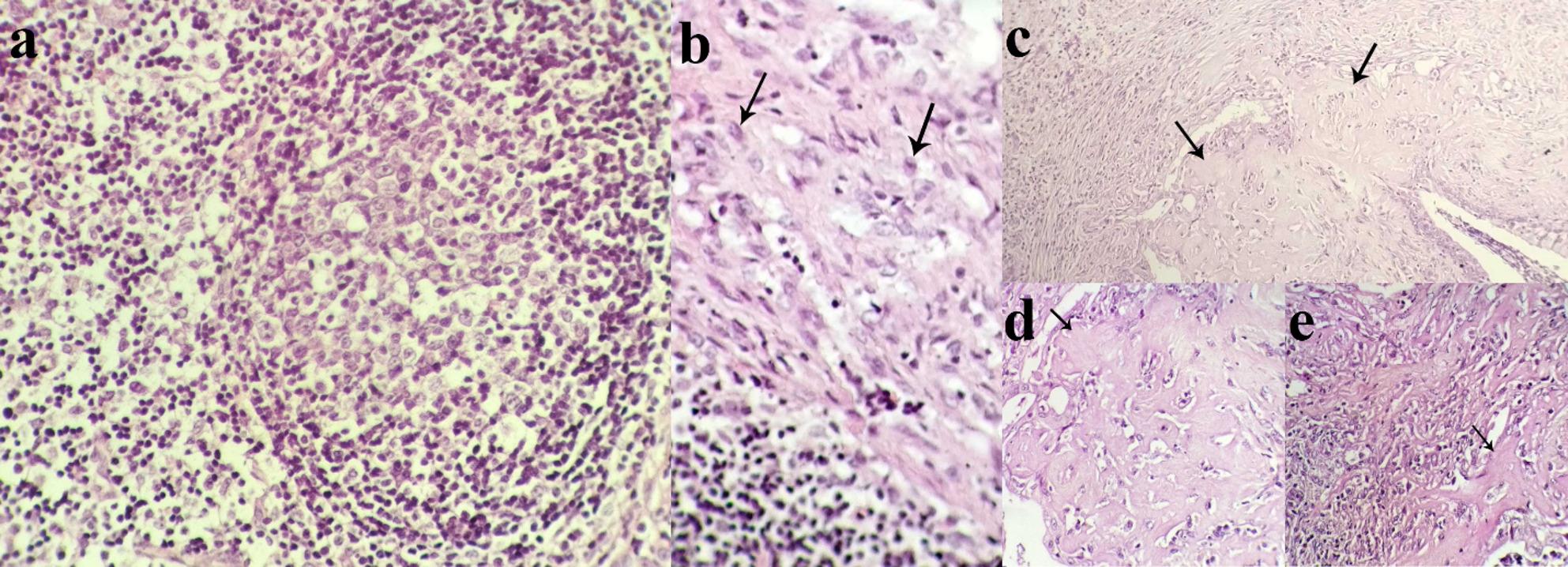
**(a **and** b)** Photomicrographs of the popliteal lymph node. **a** Hyperplasia and edema of the lymph node, **b** tumor cells. H&E stain 400X **(c**,** d**,**e)**Photomicrograph of the lung. Black arrows show metastatic foci of osteoid strands. H&E stain 40X (**c**) and 400X (**d** and **e**)

A microscopic examination of the bone tissue revealed hyperactive osteoblasts, bone remodeling, and numerous pleomorphic tumor cells with enlarged nuclei and abnormal spindle shapes. Furthermore, necrosis was present, along with numerous vascularizations, multinucleated tumor giant cells with abundant glassy eosinophilic cytoplasm, and mitotic figures (Fig. 5). Microscopic examination of the popliteal lymph node tissue revealed follicular hyperplasia-associated edema. Furthermore, metastatic spindle-like tumor cells were seen (Fig. 6). Severe bronchopneumonia, severe fibrosis with mononuclear inflammatory cell infiltration, squamous metaplasia of the bronchial epithelium, hyperplasia of the mucosal glands, and type II pneumocytes were seen in the lung tissue. There were metastatic foci of osteoid strands (Fig. 6). Histology of the liver tissue revealed centrilobular necrosis, vacuolar degeneration of hepatocytes, accumulation of hemosiderin and ceroid pigments, and the initial signs of fibrosis in the portal triads with inflammatory cell infiltration. Histology of the spleen tissue revealed degeneration of both the red and white pulp.

**Fig. 7:**
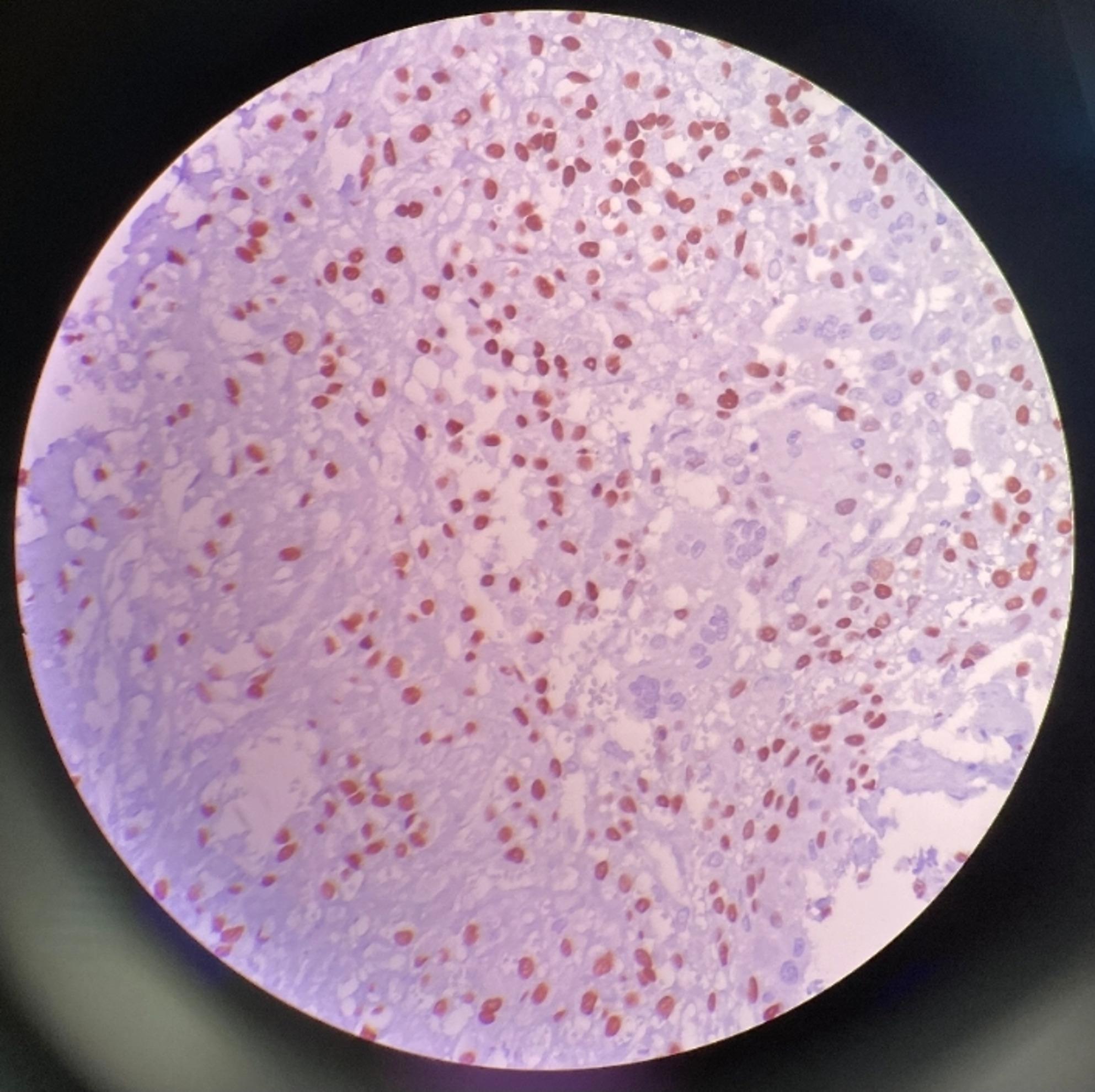
Strong nuclear positivity for SATB2 in the metatarsal lesion. 400X

To further characterize the bone lesion, immunohistochemistry was performed. The neoplastic spindle cells exhibited diffuse, strong nuclear SATB2 labeling, confirming their osteoblastic origin, although this marker is not subtype-specific (Fig. 7).

Ultimately, the case was diagnosed as a fibroblastic osteosarcoma with metastasis to the lung and lymph node, accompanied by hepatitis and splenitis.

## Discussion and conclusions

The present report describes an exceptionally aggressive case of fibroblastic osteosarcoma in a one-year-old cat, a finding that contrasts sharply with the typical demographics and biological behavior of feline osteosarcoma.

Most appendicular osteosarcomas in cats tend to progress slowly, have a low metastatic potential, and generally have a more favorable prognosis than those seen in dogs, with metastasis occurring in fewer than 10% of cases [1–3]. Conversely, the juvenile cat in this report demonstrated rapid tumor progression, early metastasis to the lungs and lymph nodes, and significant clinical decline, highlighting the aggressive characteristics of this uncommon histological subtype.

The integration of radiologic, cytologic, histopathologic, and immunohistochemical findings was critical for confirming the diagnosis. Radiographs demonstrated an aggressive bone lesion characterized by osteolysis, cortical destruction, and a Codman’s triangle periosteal reaction, consistent with a malignant bone tumor. Cytology revealed atypical osteoblasts and osteoid matrix, and histopathology identified spindle-shaped neoplastic cells forming eosinophilic osteoid, confirming the fibroblastic subtype. Immunohistochemistry served as an adjunctive tool: SATB2 demonstrated strong nuclear positivity, confirming osteoblastic lineage, but was not used to further subtype the tumor, as SATB2 cannot distinguish among the recognized variants [9, 10]. Although subtype-specific immunohistochemical markers do not exist in veterinary medicine, the constellation of morphological features was sufficient to classify the tumor as a fibroblastic osteosarcoma using established criteria.

The possibility of feline digitopulmonary syndrome, which commonly involves metastasis from primary lung tumors to the digits, was also considered. However, the osteogenic nature of the lung lesion, the absence of epithelial features in the metatarsal mass, and the histopathologic evidence of osteoid production in the lung excluded this differential diagnosis [11].

The cat’s extremely young age is a notable and unusual aspect of this report. Osteosarcoma usually affects older cats, with a median age of about 10 to 11 years [2, 3, 12, 13]. Nonetheless, infrequent cases have been reported in young animals, including individuals as young as 1 year old [14]. In this instance, the early onset of osteosarcoma suggests the potential for underlying factors. Yet, no apparent cause was detected, such as changes related to fractures, trauma, vaccines, prior surgical interventions such as Tibial Plateau Leveling Osteotomy (TPLO), enucleation, or onychectomy [15–22]. The exceptionally young age of this patient therefore highlights the possibility that, although uncommon, osteosarcoma should remain a differential diagnosis for rapidly progressive skeletal lesions even in very young cats.

The biochemical irregularities in this case included elevated cholesterol levels and increased serum ALP activity, findings observed in dogs and cats with osteosarcoma [23, 24]. There was also mild anemia, which was likely a sign of the anemia of chronic disease associated with neoplastic conditions [6]. These clinicopathological findings were indicative of tumor-associated systemic effects and further demonstrated the severity of disease progression.

Collectively, this case broadens the documented clinical spectrum of feline osteosarcoma by demonstrating that the fibroblastic subtype—though not typically considered highly metastatic in cats—can behave aggressively and metastasize early. The presence of both pulmonary and lymphatic metastasis in a juvenile patient underscores the importance of maintaining a broad differential diagnosis for rapidly progressive appendicular bone lesions in young animals. Additional studies are needed to determine whether such cases represent a rare but distinct biological subset of feline osteosarcoma or an underrecognized manifestation within the existing classification framework.

## Data Availability

No datasets were generated or analyzed during the current study.

## Abbreviations

DSH: Domestic Shorthair
CBC: Complete Blood Count
VD: Ventrodorsal
FNA: Fine Needle Aspiration
AVMA: American Veterinary Medical Association
PCV: Packed Cell Volume
HB: Hemoglobin
RBC: Red Blood Cell
MCV: Mean Corpuscular Volume
MCHC: Mean Corpuscular Hemoglobin Concentration
RDW: Red cell Distribution Width
WBC: White Blood Cell
ALP: Alkaline Phosphatase
TPLO: Tibial Plateau Leveling Osteotomy
IHC: Immunohistochemistry

## References

[1] Vail DM, Thamm DH, Liptak JM. Withrow and MacEwen’s Small Animal Clinical Oncology. 6th ed. Withrow and MacEwen’s Small Animal Clinical Oncology. Saunders; 2020. 1–842 p.

[2] Heldmann E, Anderson MA, Wagner-Mann C. Feline osteosarcoma: 145 cases (1990–1995). J Am Anim Hosp Assoc. 2000;36(6):518–21. 11105889 10.5326/15473317-36-6-518

[3] Marconato L, Annoni M, Massari F, Zanardi S, Stefanello D, Ferrari R, et al. A retrospective Italian Society of Veterinary Oncology (SIONCOV) study of 56 cats with appendicular osteosarcoma. Vet Comp Oncol. 2024;22(2):198–203. 38327132 10.1111/vco.12966

[4] Misdorp W, Hart AA. Some prognostic and epidemiologic factors in canine osteosarcoma. J Natl Cancer Inst. 1979;62(3):537–45. 283283 10.1093/jnci/62.3.537

[5] Meuten DJ. Tumors in Domestic Animals. 5th ed. Wiley-Blackwell; 2017.

[6] Weiss DJ, Wardrop KJ. Schalm’s Veterinary Hematology. Wiley; 2011.

[7] Kaneko JJ, Harvey JW, Bruss ML. Clinical Biochemistry of Domestic Animals [Internet]. Academic Press; 2008. Available from: https://books.google.com/books?id=spsD4WQbL0QC

[8] American Veterinary Medical Association. AVMA guidelines for the euthanasia of animals: 2020 edition. Schaumburg, IL; 2020.

[9] Milton S, Prabhu AJ, Titus VTK, John R, Backianathan S, Madhuri V. Special AT-rich sequence-binding protein 2 (SATB2) in the differential diagnosis of osteogenic and non-osteogenic bone and soft tissue tumors. J Pathol Transl Med. 2022;56(5):270–80. 36128863 10.4132/jptm.2022.07.11 PMC9510043

[10] Shank AMM, Snook E, Cavender K, McCoy J, Sorensen N, Siegrist B et al. Special AT-rich sequence-binding protein 2 immunohistochemistry in the diagnosis of osteosarcoma in dogs. J Comp Pathol [Internet]. 2024 Nov 1 [cited 2025 Nov 25];215:14–29. Available from: https://www.sciencedirect.com/science/article/abs/pii/S0021997524003049?via%3Dihub 10.1016/j.jcpa.2024.09.001 39368249

[11] Goldfinch N, Argyle D. Feline lung–digit syndrome: unusual metastatic patterns of primary lung tumours in cats. J Feline Med Surg. 2012;14(3):202–8. 22370862 10.1177/1098612X12439267 PMC10822433

[12] Helm J, Morris J. Musculoskeletal neoplasia: an important differential for lumps or lameness in the cat. J Feline Med Surg. 2012;14(1):43–54. 22247324 10.1177/1098612X11432826 PMC11148916

[13] Sturgess K. Notes on feline internal medicine. Second Edi. Chichester, West Sussex: Wiley Blackwell; 2013. (Notes on.).

[14] Nakano Y, Kagawa Y, Shimoyama Y, Yamagami T, Nomura K, Wakabayashi H, et al. Outcome of appendicular or scapular osteosarcoma treated by limb amputation in cats: 67 cases (1997–2018). J Am Vet Med Assoc. 2022;260(S1):S24–8. 10.2460/javma.21.04.0213 34914624

[15] Baum JI, Skinner OT, Boston SE. Fracture-associated osteosarcoma of the femur in a cat. Can Vet J. 2018;59(10):1096–8. 30510315 PMC6135274

[16] Sonnenschein B, Dickomeit MJ, Bali MS. Late-onset fracture-associated osteosarcoma in a cat. Vet Comp Orthop Traumatol. 2012;25(5):418–20. 22581024 10.3415/VCOT-11-10-0143

[17] BENNETT D, CAMPBELL JR. Osteosarcoma associated with healed fractures. J Small Anim Pract. 1979;20(1):13–8. 282453 10.1111/j.1748-5827.1979.tb07016.x

[18] Simon T, Kudnig BS. Veterinary Surgical Oncology [Internet]. 2nd ed. Wiley Blackwell; 2022. Available from: https://www.wiley.com/en-us/Veterinary+Surgical+Oncology%2C+2nd+Edition-p-9781119090229

[19] Breitreiter K. Late-onset osteosarcoma after onychectomy in a cat. J Feline Med Surg Open Rep. 2019. 10.1177/2055116919842394. 10.1177/2055116919842394 PMC6457030 31007940

[20] Groskopf BS, Dubielzig RR, Beaumont SL. Orbital extraskeletal osteosarcoma following enucleation in a cat: a case report. Vet Ophthalmol. 2010;13(3):179–83. 20500718 10.1111/j.1463-5224.2010.00774.x

[21] Woog J, Albert DM, Gonder JR, Carpenter JJ. Osteosarcoma in a phthisical feline eye. Vet Pathol. 1983;20(2):209–14. 6573055 10.1177/030098588302000208

[22] Saba C. Vaccine-associated feline sarcoma: current perspectives. Vet Med Res Rep. 2017;8:13–20. 10.2147/VMRR.S116556 PMC6042530 30050850

[23] Thrall MA, Weiser G, Allison RW, Campbell TW. Veterinary Hematology, clinical Chemistry, and cytology. Third edit. Wiley; 2022.

[24] Leeper H, Viall A, Ruaux C, Bracha S. Preliminary evaluation of serum total cholesterol concentrations in dogs with osteosarcoma. J Small Anim Pract. 2017;58(10):562–9. 28660727 10.1111/jsap.12702

